# Supplementary material for: The influence of dataset homology and a rigorous evaluation strategy on protein secondary structure prediction
Source: PLoS One. 2021 Jul 14;16(7):e0254555. doi: 10.1371/journal.pone.0254555 (PMC8279362; doi:10.1371/journal.pone.0254555)
Supplement: S1 Table — (PDF) [file pone.0254555.s001.pdf]

S1 Table. The version and parameter settings of the PSI-BLAST engine for the utilized SSP algorithms.

Algorithm	PSI-BLAST engine	PSI-BLAST parameter settings
DeepCNF, v1.02	blastpgp	-j 3 -h 0.001
Psipred, v3.3	psiblast	-num_iterations 3 -inclusion_ethresh 0.001
RaptorX, v1.0	psiblast	-j 5 -h 0.001
Scorpion, v1.0	blastpgp	Not available
Spider, v2.0 (Spider2)	blastpgp	-num_iterations 3
SpineX, v2.0	blastpgp	-j 3
SSpro8, v5.2	blastpgp	-h 1e-10 -e 0.001
MUFOLD_SS, v2.0	psiblast	-num_iterations 3 -evalue 0.001
Porter, v5.0 (Porter 5)	psiblast	-num_iterations 2 -evalue 0.001 -inclusion_ethresh 0.001 -num_alignments 300000
Spider, v3.0 (Spider3)	psiblast	-num_iterations 3 -num_alignments 1
Unified settings	psiblast (NCBI BLAST v2.6.0)	-evalue 100 -num_iterations 3 -inclusion_ethresh 0.001 -num_descriptions 500 -num_alignments 500
