# Supplementary material for: The influence of dataset homology and a rigorous evaluation strategy on protein secondary structure prediction
Source: PLoS One. 2021 Jul 14;16(7):e0254555. doi: 10.1371/journal.pone.0254555 (PMC8279362; doi:10.1371/journal.pone.0254555)
Supplement: S2 Table — (PDF) [file pone.0254555.s002.pdf]

S2 Table. Performance verification of the applied state-of-the-art SSP algorithms.

**Datasets**	**TS115 *versus* UniRef90-2015**		**CASP12 *versus* UniRef90-2015**	
**Algorithm**	**Q3**	**Q3 (reported)**	**Q3**	**Q3 (reported)**
DeepCNF	0.819	0.823[a]	0.828	0.821[a]
PSIPRED	0.801	0.802[a]	0.783	0.780[a]
RaptorX	0.807	0.812[b]	0.793	0.791[b]
Scorpion	0.815	0.817[a]	0.823	0.805[a]
Spider2	0.819	0.819[a]	0.812	0.798[a]
SpineX	0.800	0.801[a]	0.783	0.769[a]
SSpro8	0.788	0.795[b]	0.779	0.776[b]
Average	*0.807*	*0.810*	*0.800*	*0.791*
**Algorithm**	**Q8**	**Q8 (reported)**	**Q8**	**Q8 (reported)**
DeepCNF	0.703	0.720[a]	0.728	0.730[a]
SSpro8	0.671	0.666[b]	0.675	0.656[b]
RaptorX	0.698	0.697[b]	0.694	0.651[b]
Average	*0.703*	*0.694*	*0.699*	*0.679*

[a]Obtained from [44]. Query sets: TS115 and a reduced CASP12. PSSM reference set: UniRef90-2015.

[b]Obtained from [35]. Query sets: CullPDB and CASP11. PSSM reference set: UniRef90-2015.
