# Supplementary figures and images for: The influence of dataset homology and a rigorous evaluation strategy on protein secondary structure prediction
Source: PLoS One. 2021 Jul 14;16(7):e0254555. doi: 10.1371/journal.pone.0254555 (PMC8279362; doi:10.1371/journal.pone.0254555)

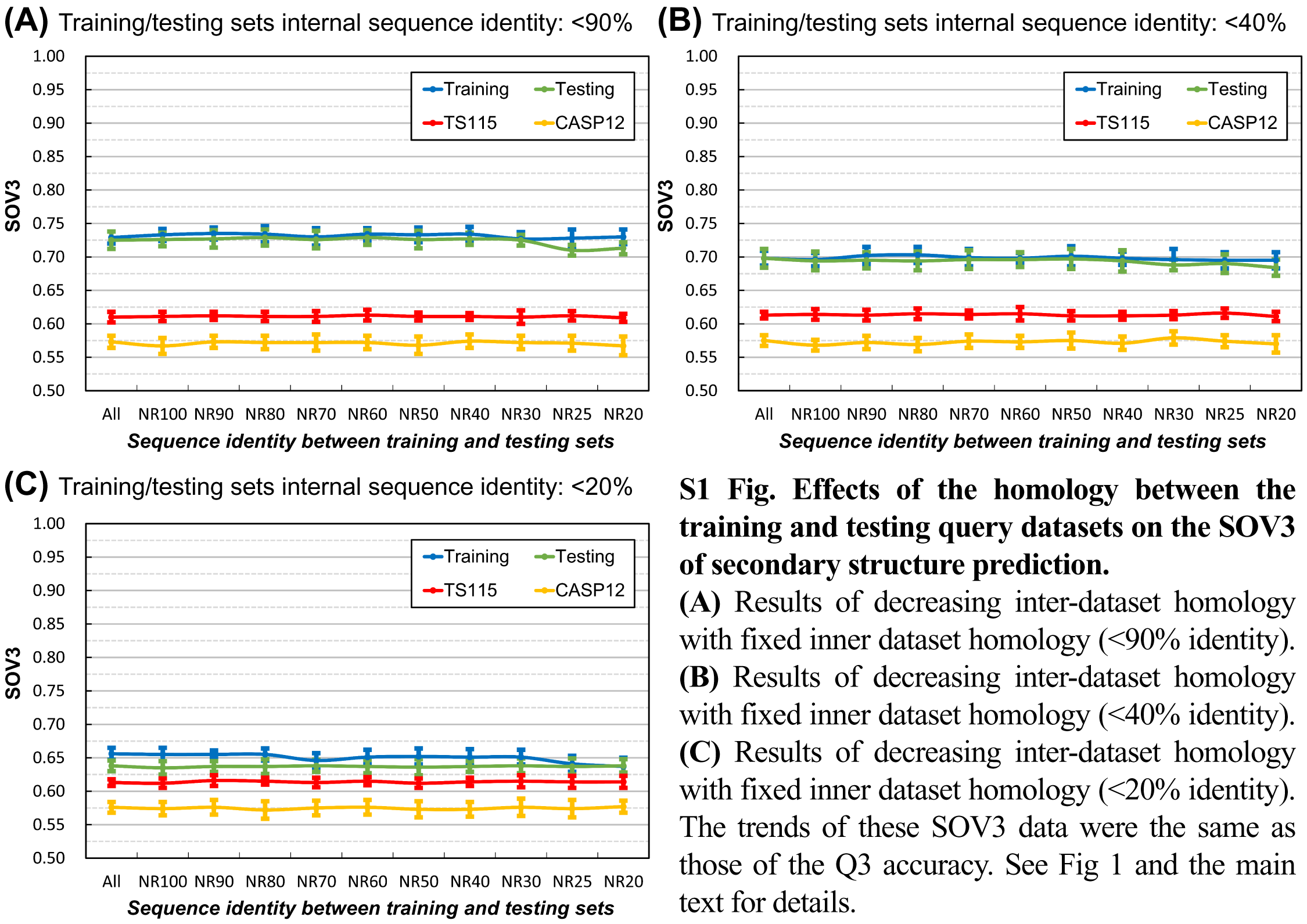

Supplement: S1 Fig — (TIF) [file pone.0254555.s003.tif]

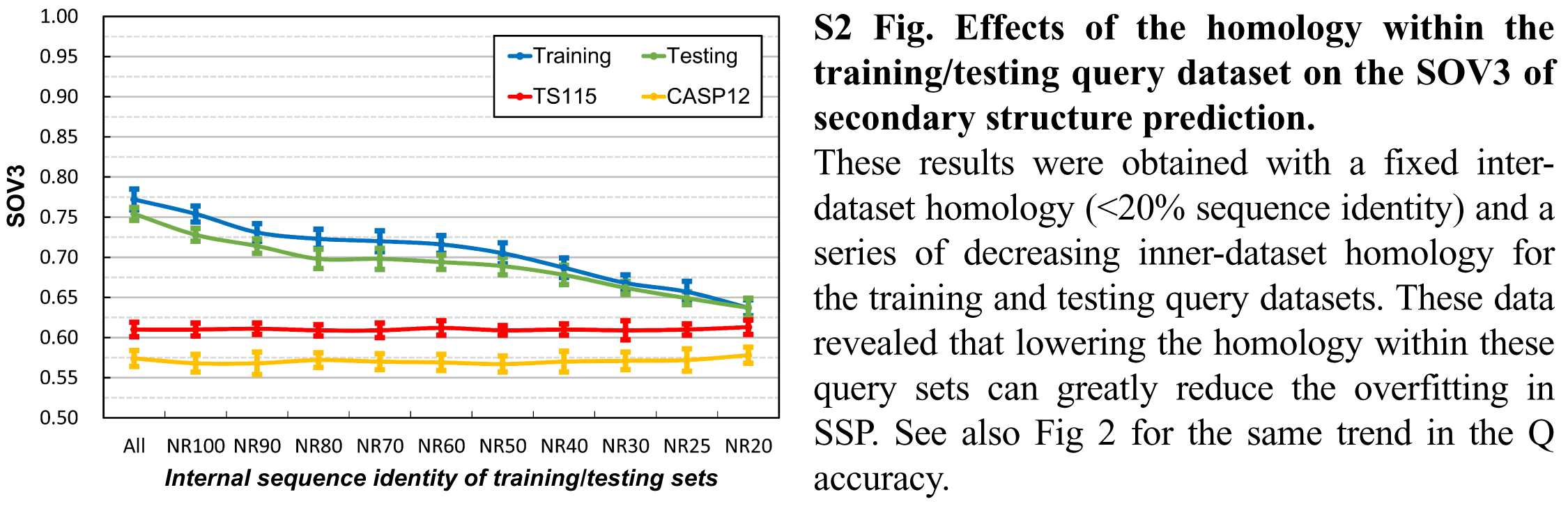

Supplement: S2 Fig — (TIF) [file pone.0254555.s004.tif]

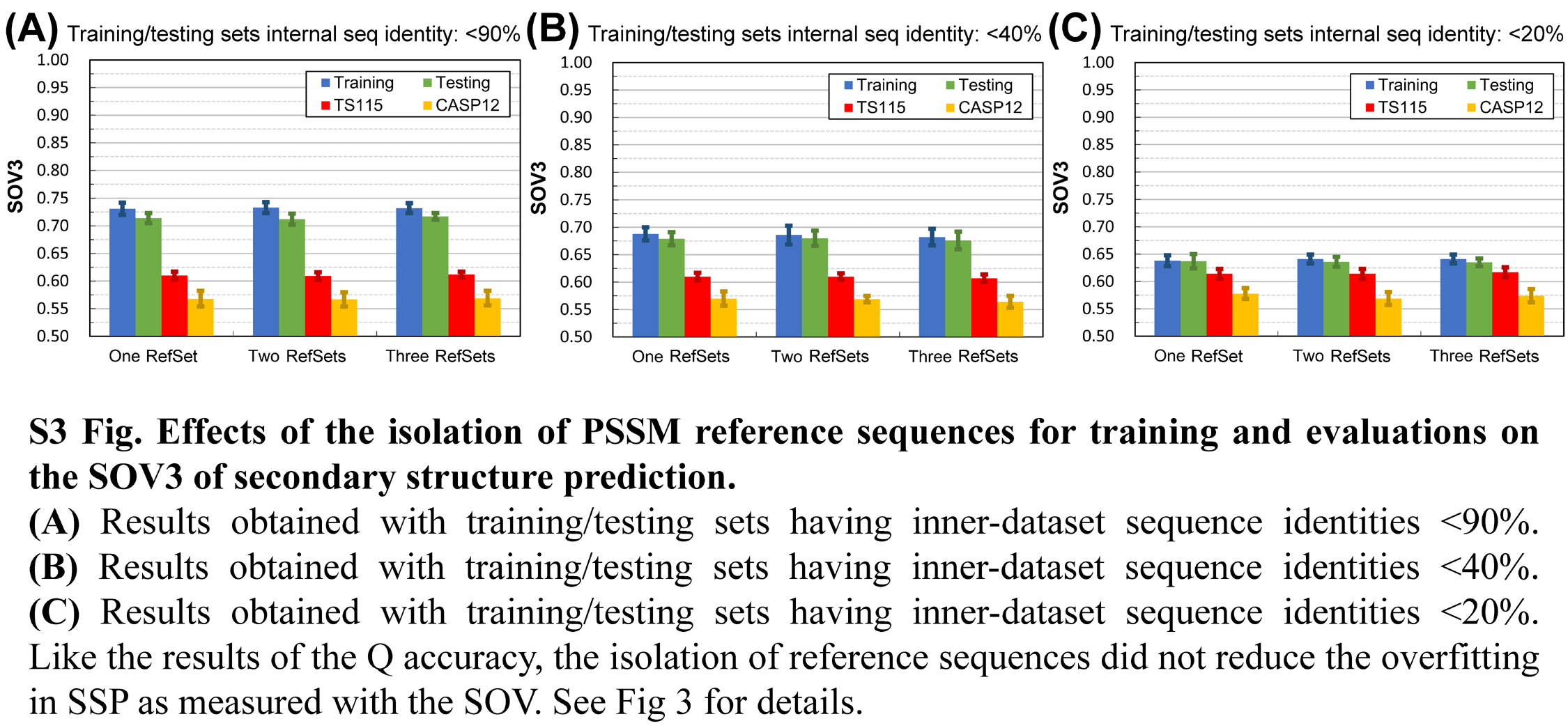

Supplement: S3 Fig — (TIF) [file pone.0254555.s005.tif]

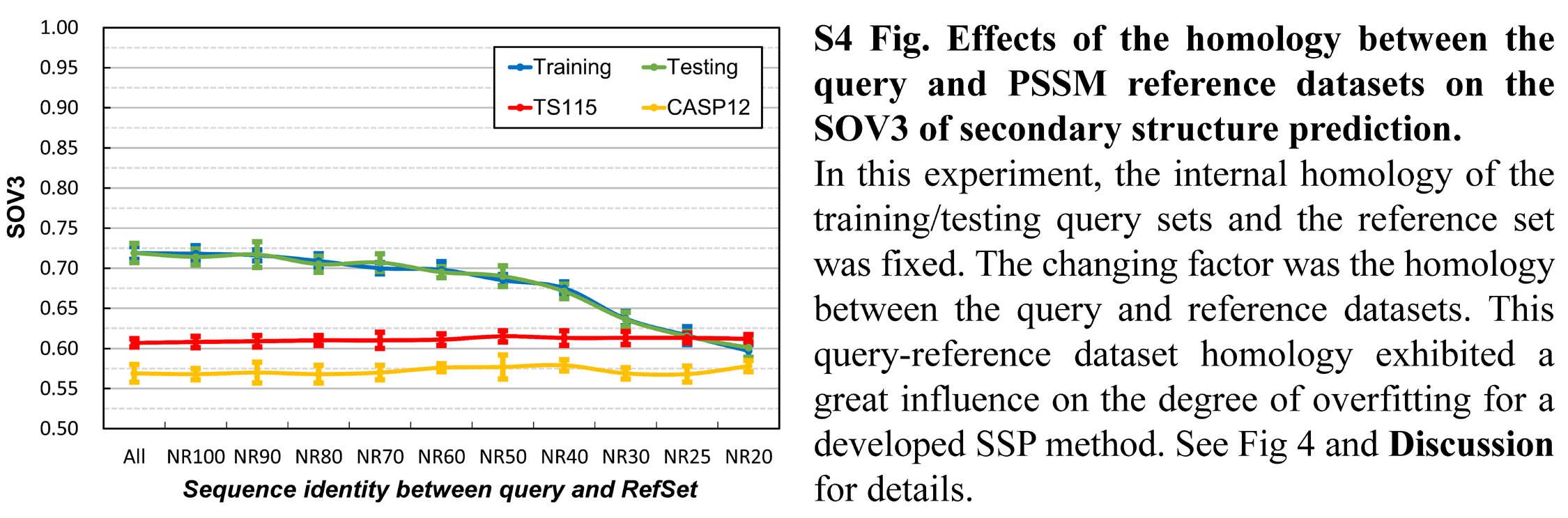

Supplement: S4 Fig — (TIF) [file pone.0254555.s006.tif]

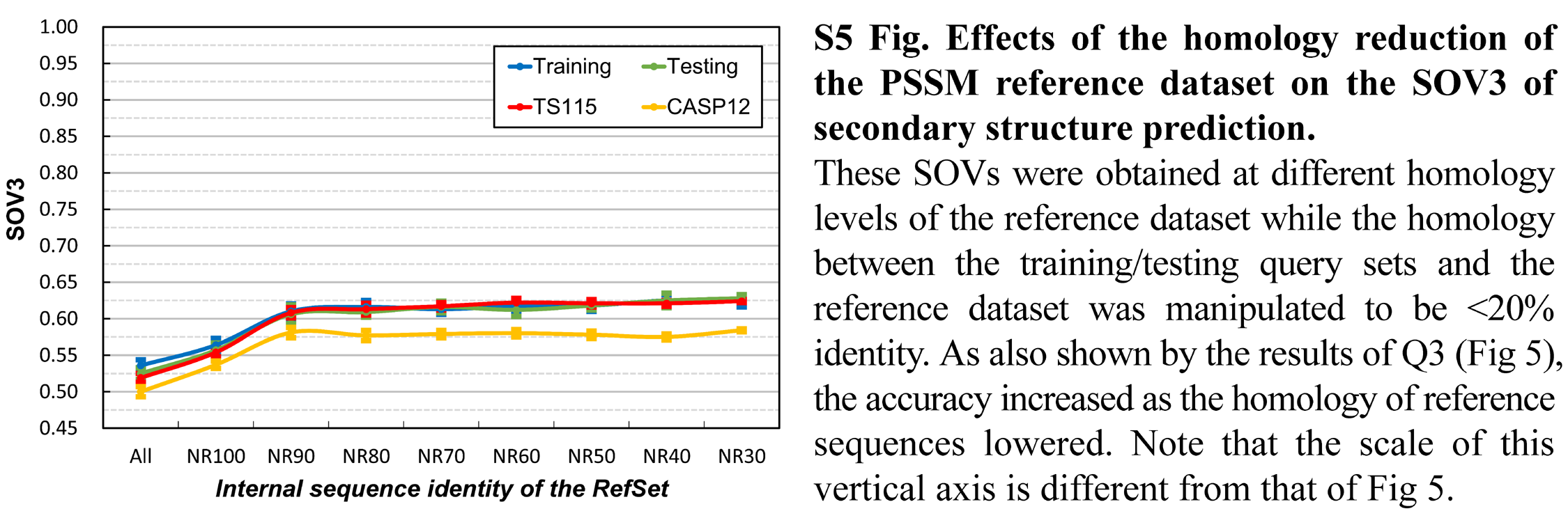

Supplement: S5 Fig — (TIF) [file pone.0254555.s007.tif]

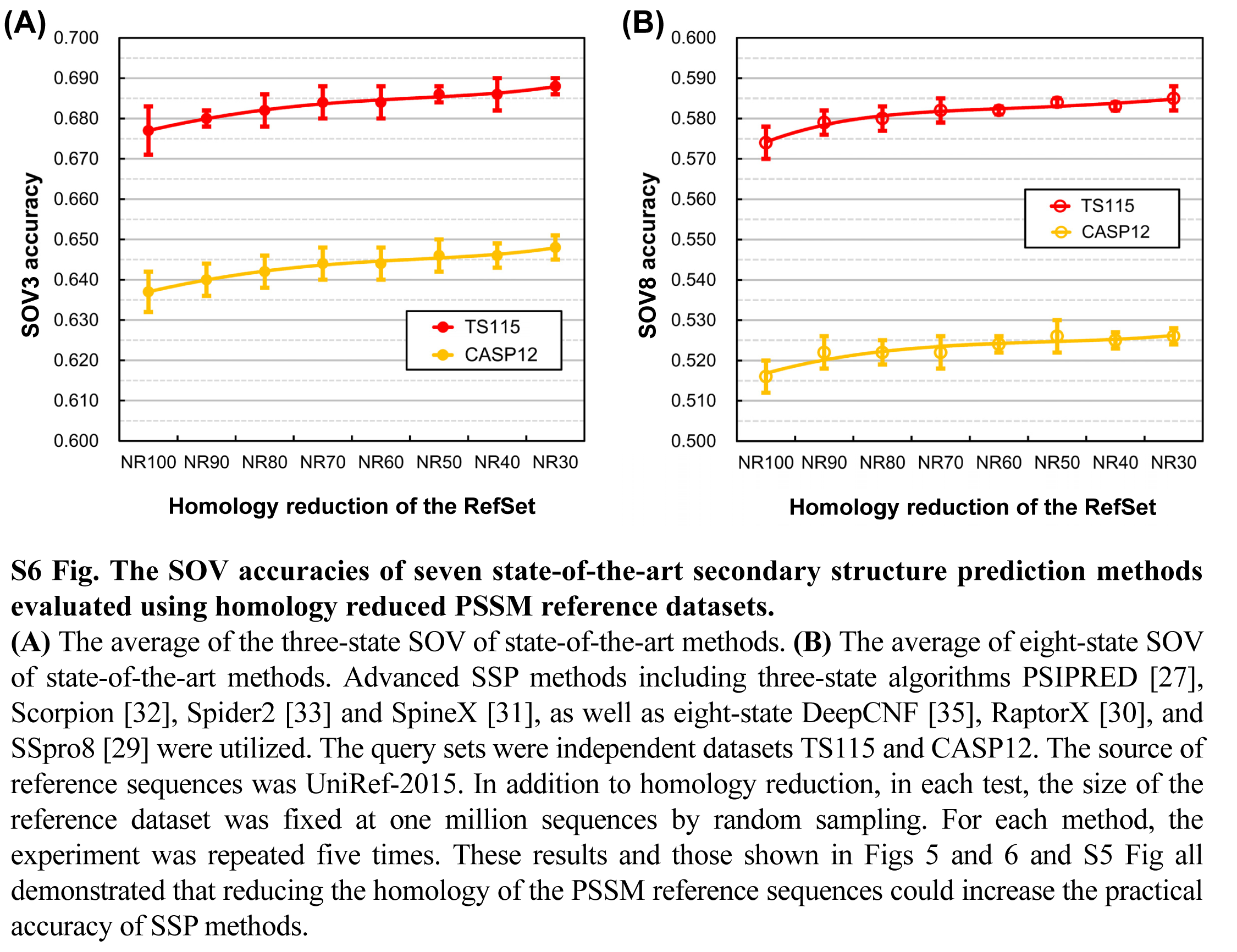

Supplement: S6 Fig — (TIF) [file pone.0254555.s008.tif]

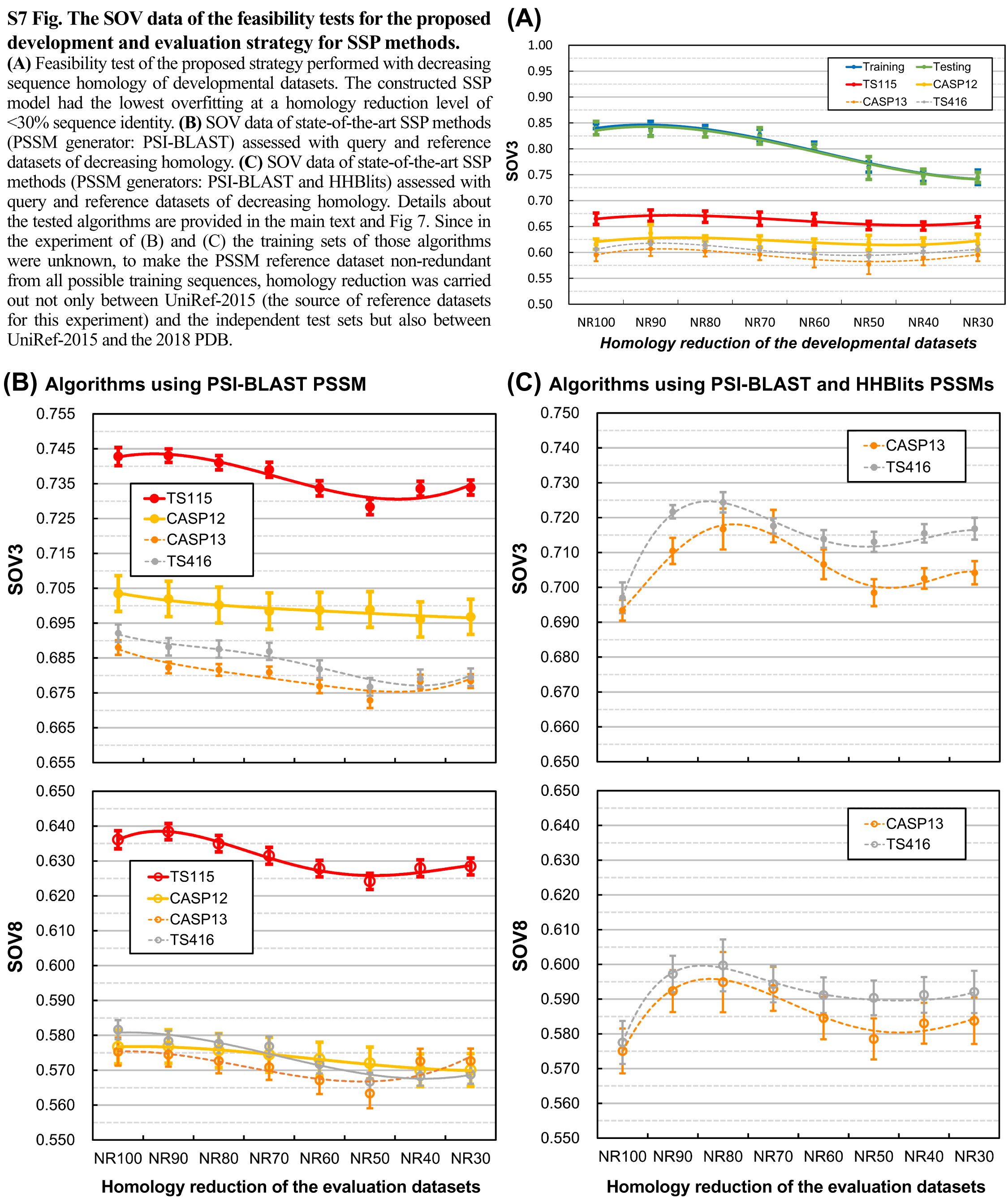

Supplement: S7 Fig — (TIF) [file pone.0254555.s009.tif]

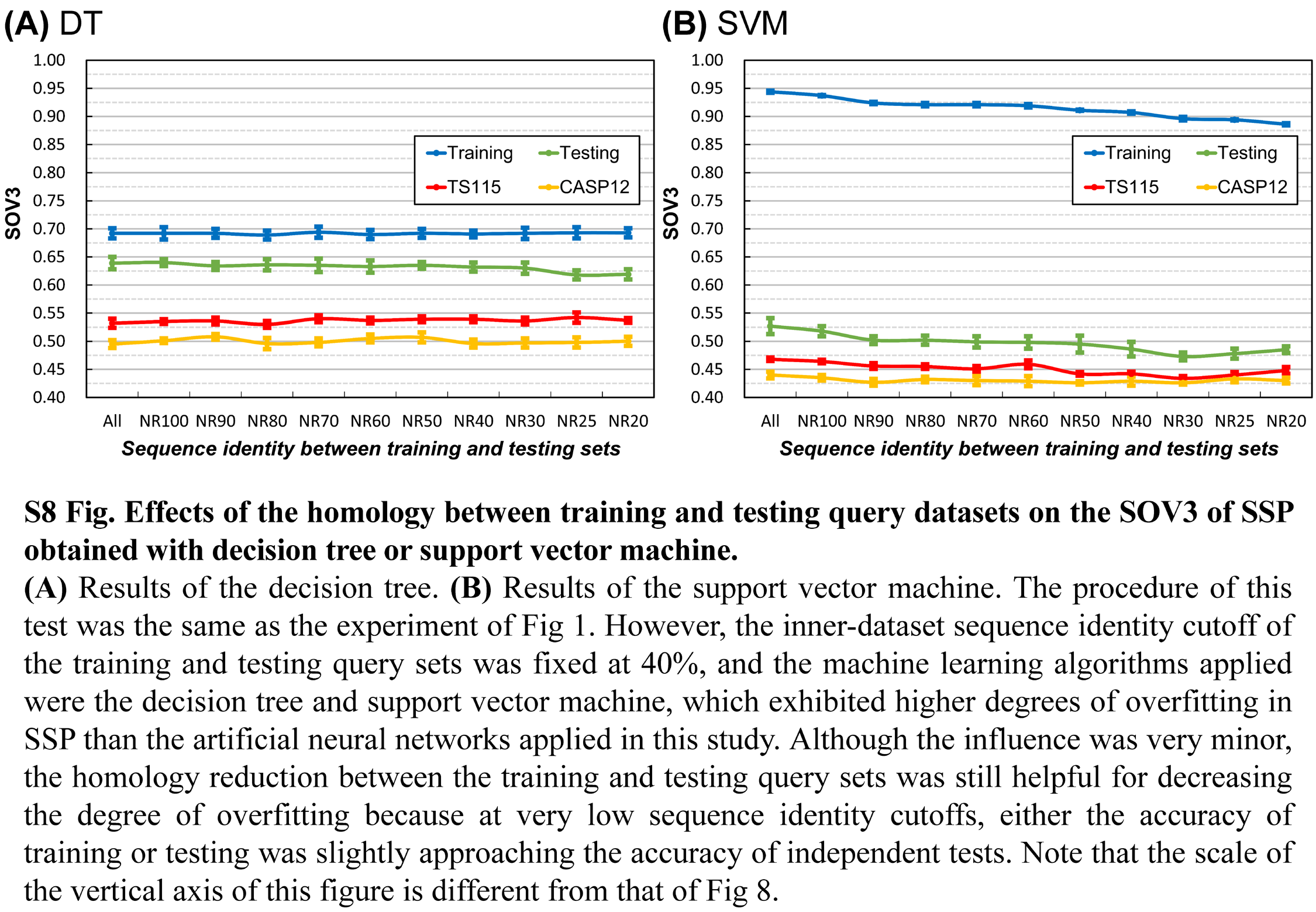

Supplement: S8 Fig — (TIF) [file pone.0254555.s010.tif]

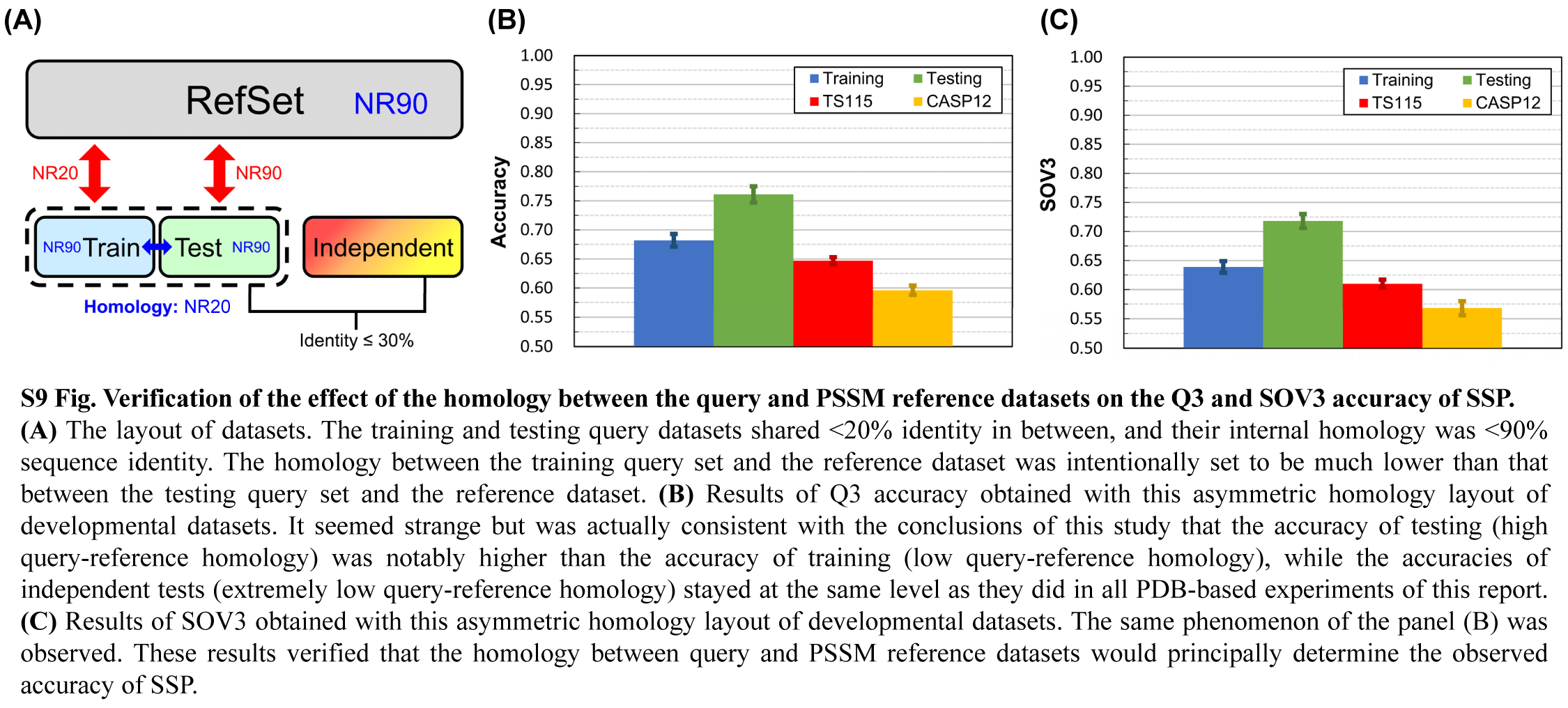

Supplement: S9 Fig — (TIF) [file pone.0254555.s011.tif]

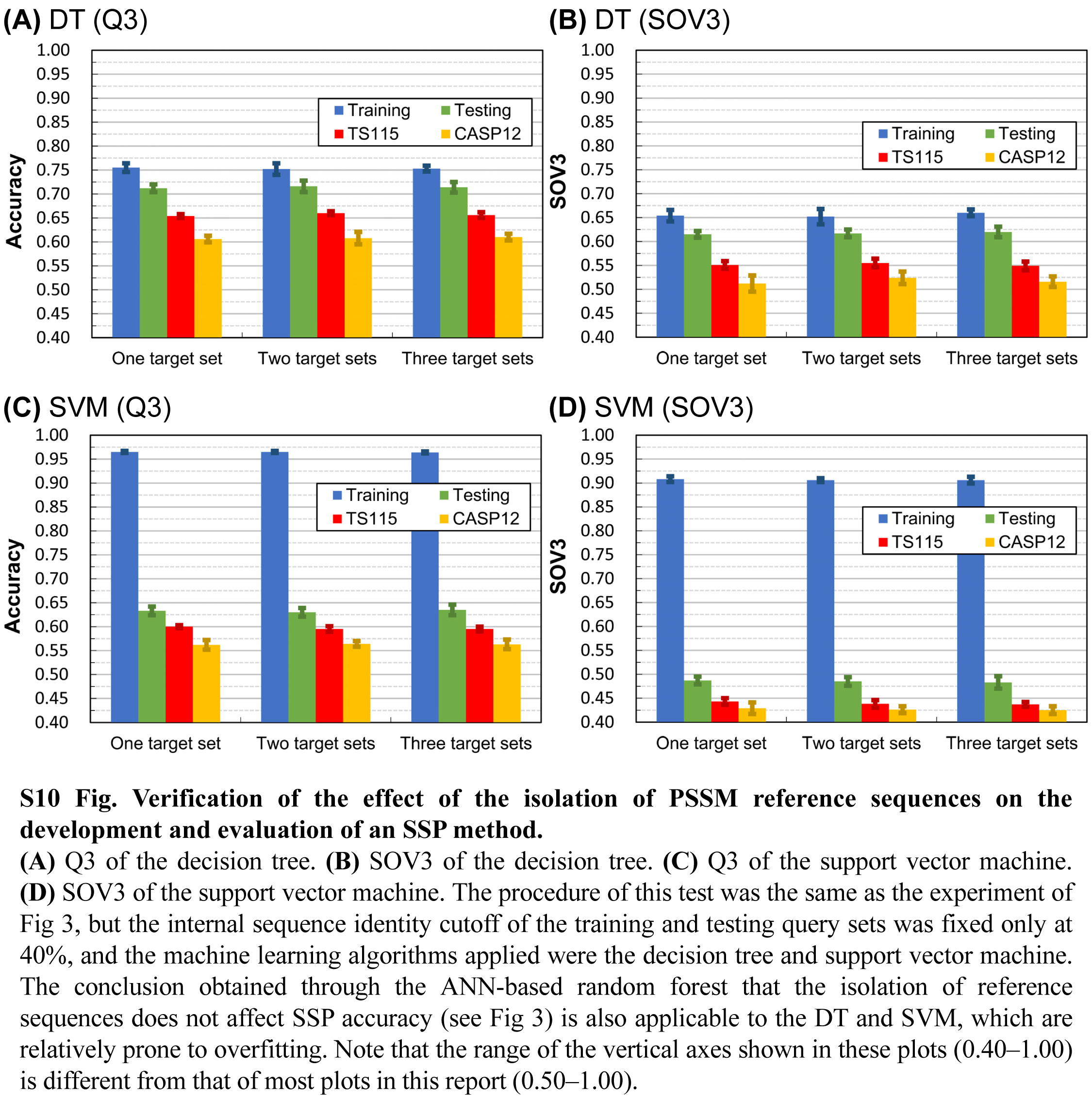

Supplement: S10 Fig — (TIF) [file pone.0254555.s012.tif]

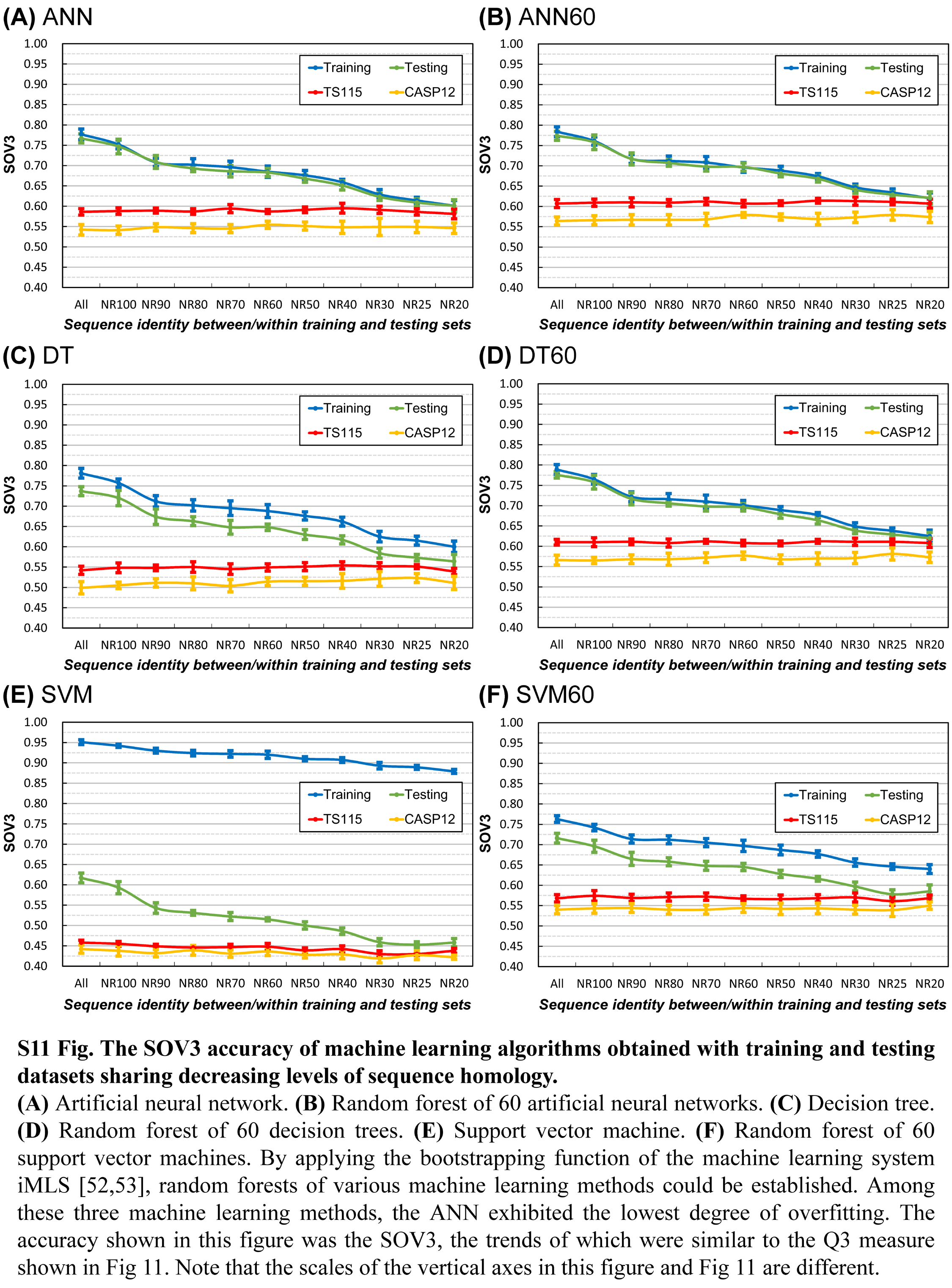

Supplement: S11 Fig — (TIF) [file pone.0254555.s013.tif]

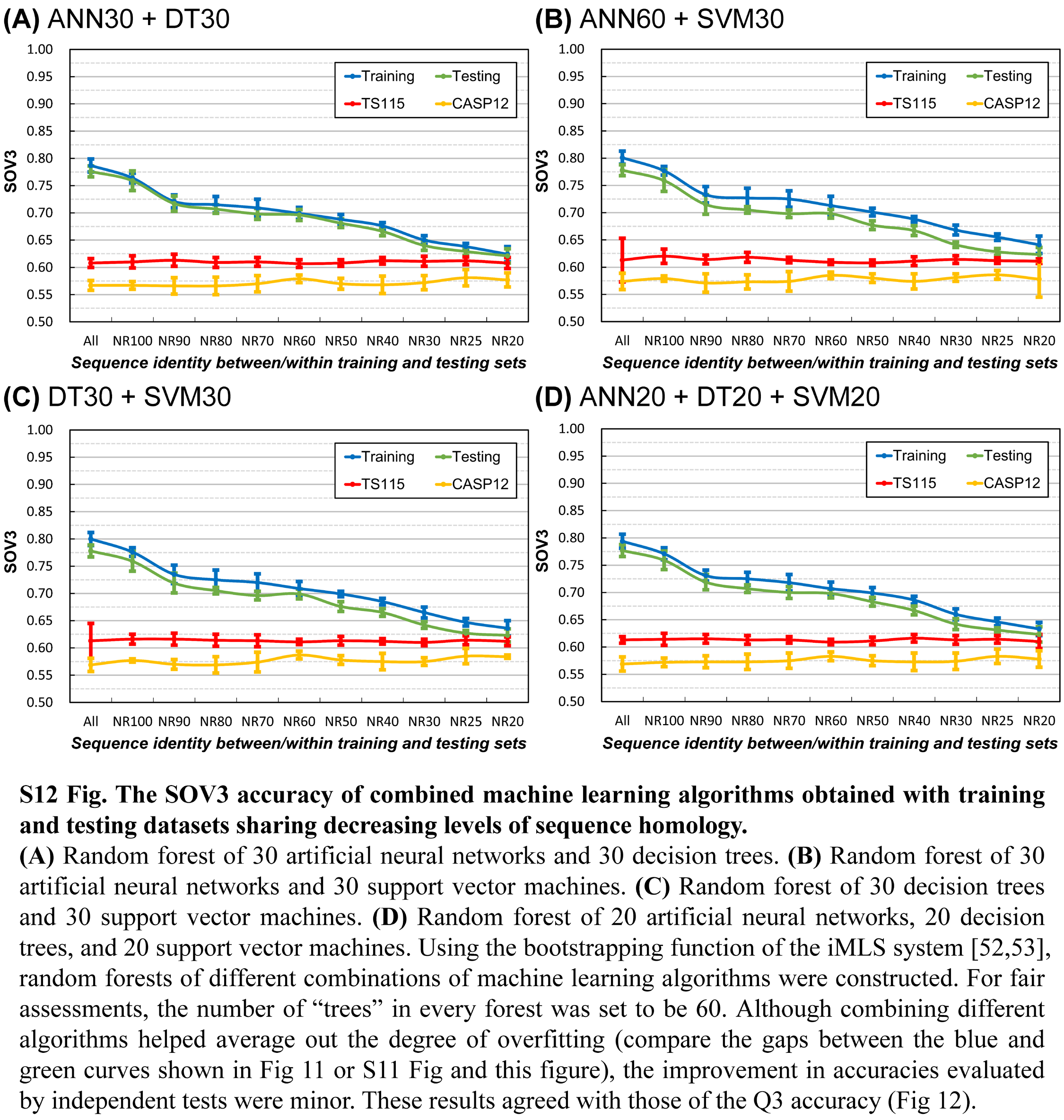

Supplement: S12 Fig — (TIF) [file pone.0254555.s014.tif]

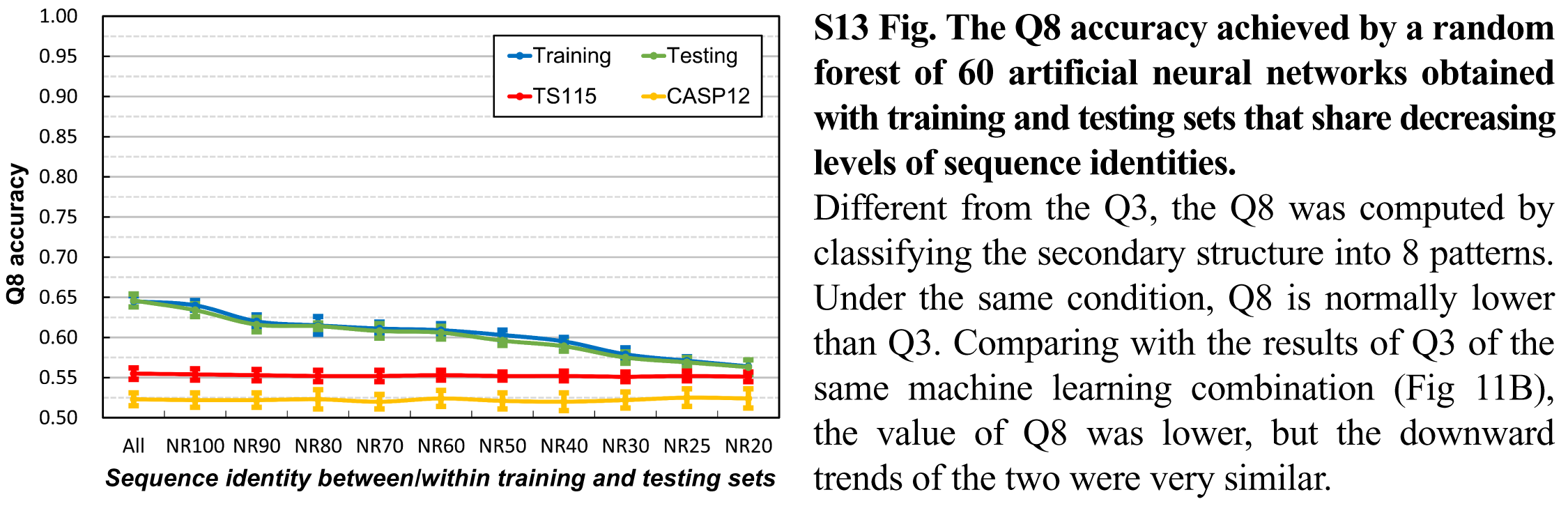

Supplement: S13 Fig — (TIF) [file pone.0254555.s015.tif]

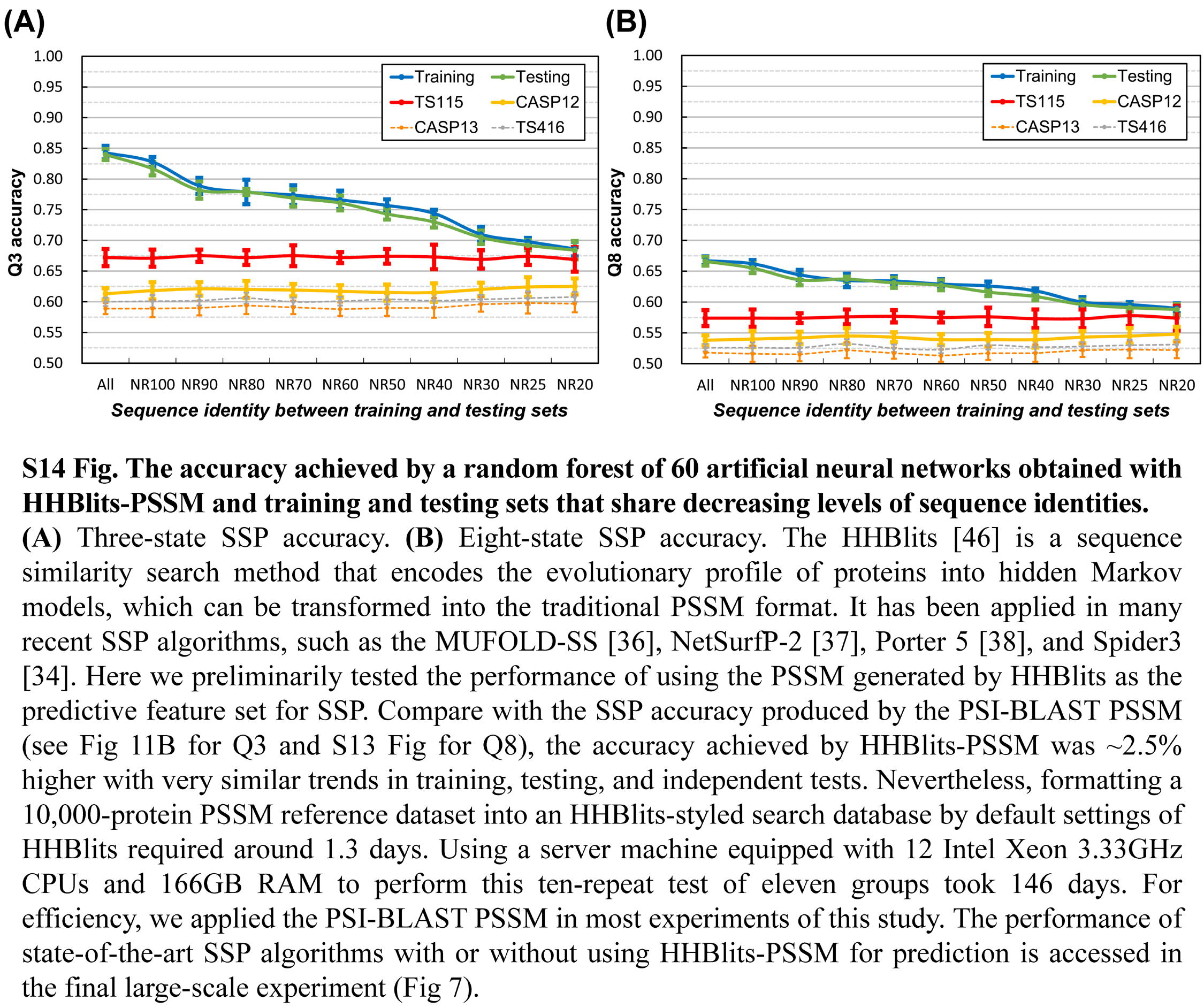

Supplement: S14 Fig — (TIF) [file pone.0254555.s016.tif]

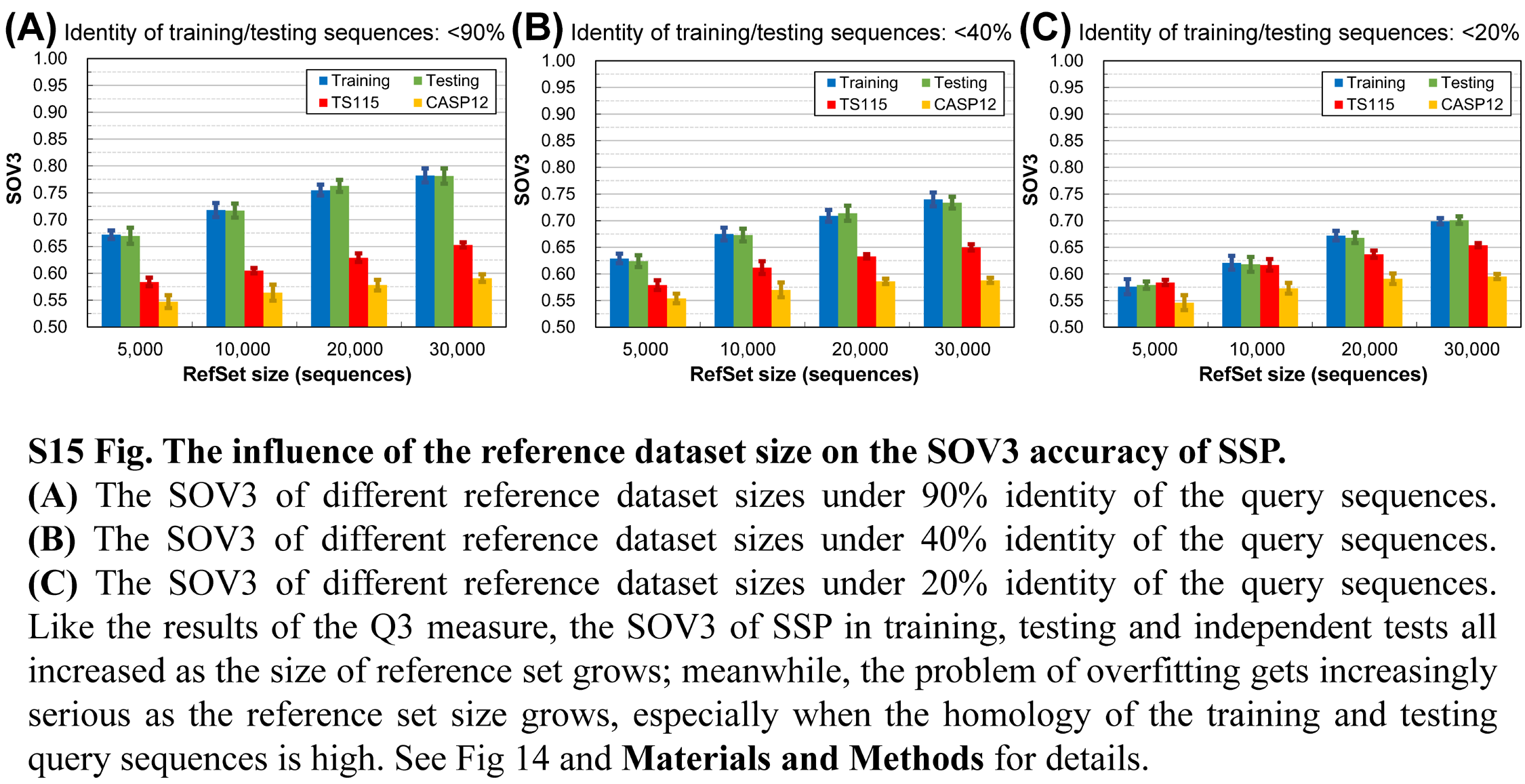

Supplement: S15 Fig — (TIF) [file pone.0254555.s017.tif]
